# Cellulose/Gold Nanocrystal Hybrids via an Ionic Liquid/Aqueous Precipitation Route

**DOI:** 10.3390/molecules14114682

**Published:** 2009-11-18

**Authors:** Zhonghao Li, Andreas Taubert

**Affiliations:** 1 Institute of Chemistry, University of Potsdam, Karl-Liebknecht-Str. 24-25, Building 26, D-14476 Golm, Germany; 2 Key Laboratory of Liquid Structure and Heredity of Materials, Ministry of Education, School of Materials Science and Engineering, Shandong University, Jinan, Shandong Province 250061, China; E-Mail: zhonghaoli@sdu.edu.cn (Z.L.); 3 Max-Planck-Institute of Colloids and Interfaces, D-14476 Golm, Germany

**Keywords:** cellulose, gold nanoparticles, ionic liquid, precipitation, hybrid material

## Abstract

Injection of a mixture of HAuCl_4_ and cellulose dissolved in the ionic liquid (IL) 1-butyl-3-methylimidazolium chloride [Bmim]Cl into aqueous NaBH_4_ leads to colloidal gold nanoparticle/cellulose hybrid precipitates. This process is a model example for a very simple and generic approach towards (noble) metal/cellulose hybrids, which could find applications in sensing, sterile filtration, or as biomaterials.

## 1. Introduction

Inorganic materials chemistry in ionic liquids (ILs) has recently attracted increasing attention [1,2,3,4,5,6,7,8]. This is because ILs, among other features, enable the synthesis of advanced materials that cannot (easily) be made in conventional solvents [9,10,11].

For well-known reasons [12,13], gold particles are among the best-studied particles in materials science. There are also several examples of gold particle synthesis in ILs [14,15,16,17,18]. Like in classical synthesis protocols, reduction of Au(III) in ILs is often achieved with various reducing agents such as NaBH_4_. Moreover, it has recently been shown that the solubility of cellulose in ionic liquids [19,20] can be exploited for the synthesis of gold microcrystals [18]. 

The current study shows that injection of a mixture of HAuCl_4_ and cellulose dissolved in the IL 1-butyl-3-methylimidazolium chloride, [Bmim]Cl, into aqueous NaBH_4_ leads to gold nanoparticle/cellulose hybrids. The NaBH_4_ rapidly reduces Au(III) to Au(0). At the same time, cellulose, which is soluble in [Bmim]Cl but not water, precipitates as fine nanofibers. The process is thus a model approach for the synthesis of (noble) metal/cellulose hybrids, which could find applications in sensing, sterile filtration, or biomaterials.

## 2. Results and Discussion

### 2.1. Results

We have investigated the effects of gold and cellulose concentration. As neither of them has a strong effect on the structure of the resulting products, in the following we will focus on one main example, which was grown with 20 mg of HAuCl_4_ and 20 mg of cellulose/g IL. The one effect that is important to control is the stirring time after injection. Immediate isolation of the products right after injection leads to poorly defined, unstructured materials (data not shown). 

**Figure 1:**
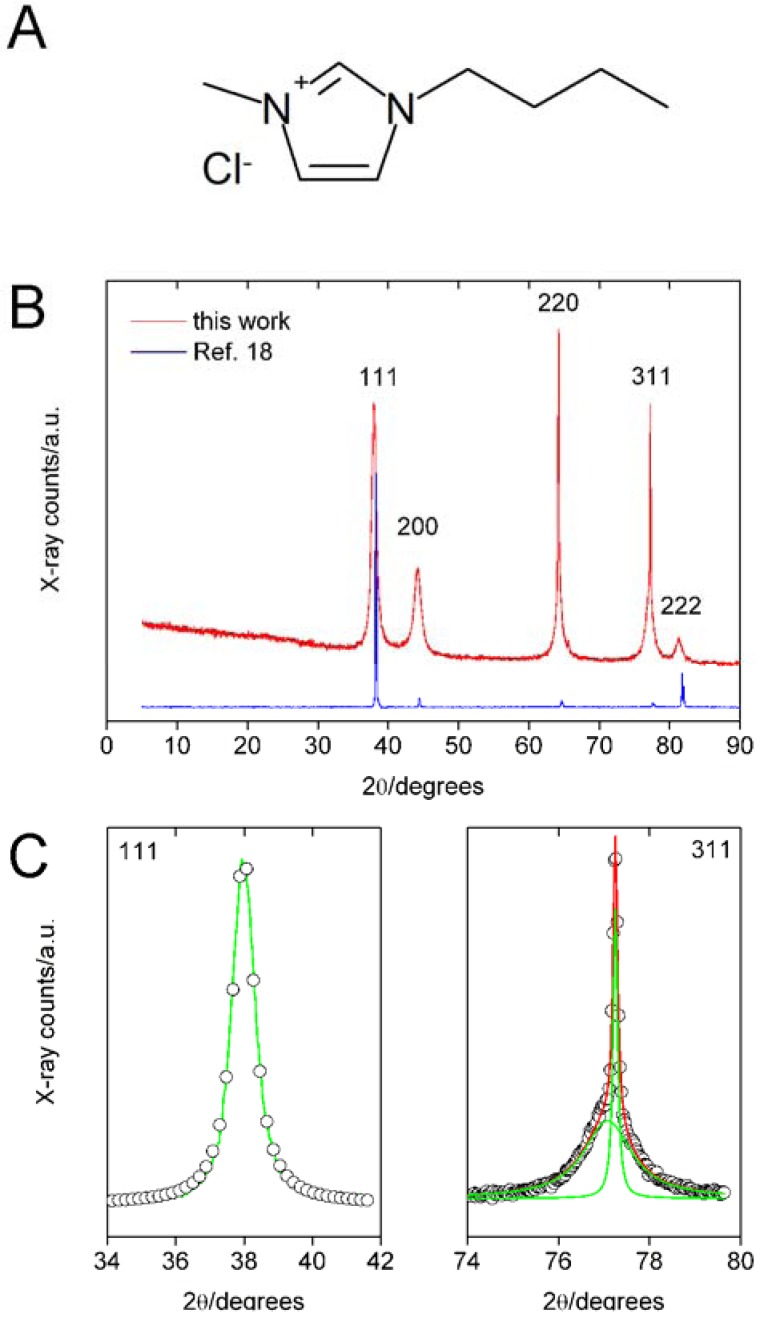
A: Structure of [Bmim]Cl. B: XRD pattern of a sample prepared via injecting a solution 20 mg of cellulose and 20 mg of HAuCl_4_·3H_2_O in 1 g of [Bmim]Cl into aqueous NaBH_4_. The pattern from ref. [18] is displayed for comparison. C: magnified (111) and (311) reflections showing the superposition of two Lorentzians for (311). Symbols are experimental data, green lines are individual Lorentzians, and red line is the superposition.

Figure 1 shows a typical X-ray diffraction (XRD) pattern of a gold nanoparticle/cellulose hybrid precipitate after isolation and drying. All products are pure face centered cubic (fcc) gold (JCPDS 04-0784). In most cases, the full widths at half maximum (FWHM) are larger than 1 degree 2θ. The (110), (200), and (222) reflections can be fitted with a single Lorentzian. The (220) and (311) can only be fitted with a superposition of two Lorentzians. The crystallite sizes D_hkl_ (coherence lengths) calculated via the Scherrer equation [24] are in all cases around 10 nm. For the sample shown in Figure 1, D_111_ is 11.2, D_200_ is 6.6, D_220_ is 7.5, D_311_ is 7.8, and D_222_ is 7.1 nm. The reflections in XRD are thus much broader than those of samples grown in [Bmim]Cl, where cellulose acted as the reducing agent and the template for gold particle formation [18]. 

Although the synthesis process of the current material and the material described in ref. [18] is quite different, XRD shows no cellulose reflections in either case. The absence of cellulose reflections indicates that the cellulose, although precipitated, is only poorly crystalline. Possibly the NaBH_4_ reducing agent not only reduces Au(III) to Au(0), but some reaction also takes place between the reducing agent and the cellulose. 

**Figure 2:**
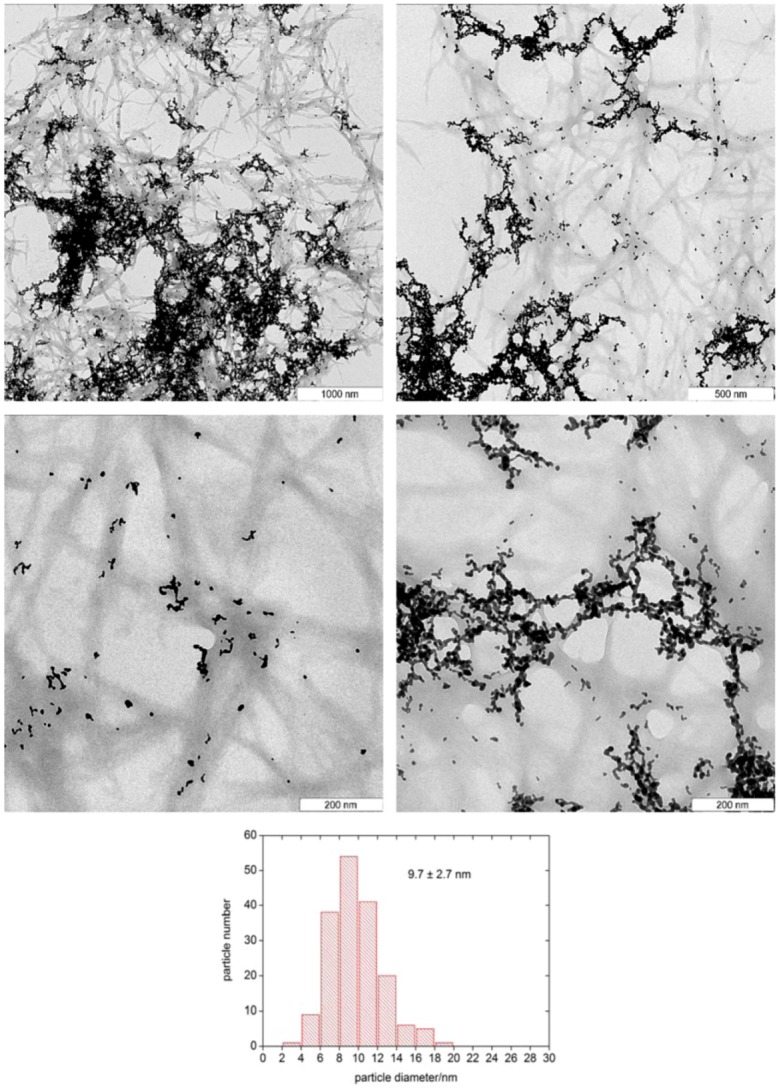
TEM images and gold particlesize distribution of the sample shown in Figure 1.

Figure 2 shows representative transmission electron microscopy (TEM) images of the same precipitate. The cellulose fibers are short and only have a length of ca. one micron. The diameter distribution is rather broad and ranges from a few nm to tens of nm. As a result, TEM proves that not only gold nanoparticles, but also cellulose precipitates, even though no cellulose reflection appears in XRD. As the fibers are rather short, TEM supports the above hypothesis that, possibly, NaBH_4_ also reacts with the cellulose. Partial degradation and poor order in the cellulose fibers due to rapid precipitation could also account for the fact that XRD does not detect cellulose reflections. 

TEM further supports XRD, as both techniques observe gold particles with sizes below 20 nm. The D_hkl_ values from XRD agree well with the mean particle diameter of 9.7 ± 2.7 nm obtained from TEM. This suggests that the individual particles are single crystals, although TEM shows that they do not precipitate as single particles. Mostly, they form extended networks, similar to gold particles grown in polymer microgels [25,26]. The particles are exclusively observed on the cellulose fibers and individual gold wires follow the cellulose fibers. This suggests that the cellulose fibers act as scaffolds for nanoparticle growth or aggregation. The details of the process are currently under investigation. 

### 2.2. Discussion

The current paper shows that cellulose/gold(III) solutions in the IL [Bmim]Cl can be used for the fabrication of cellulose fiber/gold nanoparticle hybrid materials. The process is simple, robust, and fast and the resulting materials could be interesting for a variety of applications such as cellulose-supported gold nanoparticle sensors or for low temperature heterogeneous catalysts. The gold particles form exclusively on the cellulose fibers and often, complex particle morphologies resembling earlier examples grown in polymer microgels [25,26], are observed in the TEM. XRD only shows signals from the gold and no cellulose reflections have been observed. 

The absence of cellulose reflections indicates that the cellulose, although precipitated, is most likely only very poorly crystalline. It is also possible that the reducing agent used in this study, NaBH_4_, not only reduces the Au(III) to Au(0) nanoparticles, but it is likely that some reaction also takes place between the reducing agent and the cellulose. This assumption is further supported by the observation of rather short cellulose fibers (around 1 μm in length), which is much shorter than what is usually observed in reconstituted cellulose.

Finally, as the IL is water soluble, there is only one liquid phase after injection of the IL solution into the aqueous phase. The cellulose/gold hybrid material precipitates and the IL, residual reducing agent, and the water form a homogeneous phase, which can easily be washed out. The aqueous phase after injection did not show any color, implying that the reduction reaction of the Au(III) to Au(0) is nearly quantitative.

## 3. Experimental

### 3.1. Synthesis

Cellulose (Acros, 10 to 100 mg) and HAuCl_4_·3H_2_O (Sigma, 10 to 100 mg) were mixed with [Bmim]Cl (Acros, 1 g). The mixture was heated to 100 ºC until a clear solution formed. The solution was injected at room temperature into 0.2 M aqueous NaBH_4_ (10 mL) under stirring, which was continued for 10 minutes. The precipitated products were recovered by repeated centrifugation, washed with water and ethanol and dried at 60 °C for 5 hours. 

### 3.2. Characterization

X-ray diffraction (XRD) was done on a Nonius PDS 120 with a position sensitive detector (1 to 120 degrees 2θ) using CuKα radiation. Estimation of particle sizes was done as described previously [21,22,23]. After background subtraction, the peaks were fitted individually with Lorentzian profiles yielding the full width at half maximum (FWHM) and the x-centered position. The peaks were analyzed using the Scherrer formula [24]:

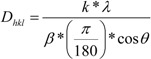

where D_hkl_ is the coherence length of the crystalline domain perpendicular to the respective hkl plane, k is a constant (here 0.9), λ is the wavelength of CuK_α_ radiation (1.5408 Å), β is the background-corrected line broadening in degrees, (π/180) is a correction factor to calculate β in radians, and θ is the scattering angle. Transmission electron microscopy (TEM) was done on a LEO 912 Omega operated at 120 kV. Samples were suspended in ethanol and a drop of the suspension was directly deposited on carbon-coated copper grids. For comparison, samples were also studied without centrifugation and washing. Image analysis was performed via AdobePhotoshop and OriginLab Origin 6.1. 

## 4. Conclusions

In summary, we show that the unique ability of ILs to dissolve cellulose can be exploited for the fabrication of cellulose nanofiber/gold nanoparticle hybrids. The process is simple, robust, and fast and the resulting materials could be interesting for applications as cellulose-supported gold nanoparticle sensors or low temperature heterogeneous catalysts.
